# High Hepatitis A Virus Seroprevalence and Low Self-Reported Vaccination Coverage Among Waste Collection Workers in Sardinia, Italy: A Retrospective Cross-Sectional Study

**DOI:** 10.3390/vaccines14090767

**Published:** 2026-09-02

**Authors:** Sara Maria Pani, Luigi Isaia Lecca, Sergio Pili, Ilaria Pilia, Vitalba Milazzo, Marcello Campagna

**Affiliations:** Department of Medical Sciences and Public Health, University of Cagliari, 09042 Monserrato, CA, Italy; saram.pani@unica.it (S.M.P.); luigii.lecca@unica.it (L.I.L.); drssa.pilia@gmail.com (I.P.); vitalbamilazzo@yahoo.it (V.M.); mcampagna@unica.it (M.C.)

**Keywords:** hepatitis A virus, HAV seroprevalence, hepatitis A vaccination, self-reported vaccination status, waste collection workers, public health

## Abstract

Background/Objectives: Hepatitis A virus (HAV) infection is a relevant biological risk in some occupational settings and has long been discussed as an occupational health concern among municipal waste collection workers. Data on HAV seroprevalence and vaccination history in this occupational group remain limited in Italy. This study investigated HAV seroprevalence, self-reported vaccination history, and factors associated with HAV seropositivity among waste collection workers in Sardinia. Methods: This retrospective cross-sectional study analyzed HAV seroprevalence and self-reported vaccination history among 326 workers in Sardinia, Italy, using occupational health surveillance data (2017–2024). Recorded variables included age, sex, smoking habits, years of service, work setting, vaccination status, and serological results. Chi-square and Mann–Whitney U tests assessed group differences; logistic regression identified factors independently associated with HAV seropositivity. Results: HAV seroprevalence was 44.2%; only 0.9% of participants reported previous HAV vaccination. Multivariable analysis identified older age, but not years of service, as independently associated with HAV seropositivity once age was modeled as a continuous variable—a pattern more consistent with birth-cohort differences in lifetime HAV exposure than with cumulative occupational exposure. Worksite 7 showed a higher prevalence ratio of HAV seropositivity relative to the reference site, but this finding was based on a small subsample and should be considered exploratory; observed worksite-level differences may reflect organizational rather than geographic factors. Conclusions: Older age, rather than occupational tenure, was independently associated with HAV seropositivity among Sardinian waste workers, a pattern consistent with birth-cohort differences in lifetime exposure. The high seroprevalence and very low vaccination uptake highlight the need to strengthen prevention through improved vaccination access, awareness, and better integration of occupational and public health strategies.

## 1. Introduction

Hepatitis A virus (HAV) is a non-enveloped RNA virus of the Picornaviridae family, primarily transmitted via the fecal–oral route [1]. Although HAV infection is generally self-limiting, it poses significant public health challenges, especially in occupational settings with potential exposure to contaminated materials, such as municipal waste collection workers [2].

In 2022, a total of 4548 hepatitis A cases were reported across 30 European Union/European Economic Area (EU/EEA) countries, corresponding to a notification rate of 1.0 per 100,000 population. The highest rates were observed in Hungary, Croatia, Romania, and Bulgaria. Overall, the data reflect a stable trend compared to 2020–2021, but lower than pre-pandemic levels [3]. In Italy, HAV incidence has declined in recent decades due to improved sanitation and vaccination strategies. However, certain occupational groups remain at elevated risk, such as municipal waste collection workers. In 2024, the SEIEVA (Integrated Epidemiological Surveillance System for Acute Viral Hepatitis), coordinated by the Italian National Health Institute, reported 443 cases of acute HAV, with an incidence of 0.85 per 100,000 inhabitants—a slight increase from 0.51 in 2023—confirming Italy’s classification as a low-endemic country [4]. The highest number of cases was registered in Lombardy, Tuscany, Emilia Romagna, and Lazio; the most affected age groups were 25–34 and 35–54 years (both with 21.9%); male individuals accounted for 60.3% of the reported cases. Notably, it has been estimated that 208 out of the 443 HAV cases reported in Italy in 2024 (47.0%) could have been prevented through vaccination [4].

Although the hepatitis A vaccine is recommended for high-risk groups, it is not included in mandatory vaccination programs in Italy [5,6] and most European countries, and vaccination coverage data are either unavailable or often fragmented and limited to specific populations, such as children in high-risk regions or travelers to endemic areas. Specifically, in Italy, vaccination against tetanus is mandatory for municipal solid waste workers according to Italian Law No. 292/1963. Conversely, immunizations against hepatitis A (HAV), hepatitis B (HBV), and typhoid fever are strongly recommended for waste and sewage handlers by the National Immunization Prevention Plan (PNPV) and the Occupational Health and Safety Decree (D.Lgs. 81/2008). Vaccination against HAV is commercially available (as monovalent or combined HAV/HBV vaccines) and provided free of charge to exposed workers, fully covered by the employer under occupational health surveillance or through the National Health Service. Similarly, the vaccine is provided free of charge to other identified high-risk populations, such as individuals with chronic liver diseases, men who have sex with men (MSM), and travelers to endemic areas. Conversely, individuals outside these designated risk categories who voluntarily choose to get vaccinated must purchase the vaccine out-of-pocket. Vaccination is primarily administered through public local health authority (ASL) immunization clinics, Travel Medicine centers, and directly within workplace facilities by occupational health services.

Despite its availability and free provision for high-risk cohorts, national HAV vaccination coverage in Italy is not routinely tracked in a centralized occupational registry; however, epidemiological studies report suboptimal coverage rates (generally under 20–30%) among waste handling cohorts, highlighting a persistent gap between occupational health recommendations and actual vaccine uptake. Several barriers hinder optimal coverage, including a low perceived risk of infection among both workers and employers, logistical challenges in scheduling vaccinations during working hours, and the lack of integrated occupational immunization registries. Conversely, key facilitators include the implementation of workplace-based (on-site) vaccination campaigns, active promotion by occupational physicians during mandatory annual health surveillance, and the integration of pre-vaccination serological screening. Therefore, documenting local HAV seroprevalence and coverage data in occupational settings is essential to inform evidence-based public health policies, overcome existing barriers, and optimize targeted intervention strategies for this high-risk workforce.

The main risk factors identified among the Italian infected subjects in 2024 included the consumption of raw or undercooked shellfish, travels to endemic areas, sexual intercourse between men, and berry consumption [4]. Furthermore, several occupational groups are at increased risk of HAV infection, including daycare personnel, healthcare workers, laboratory staff, food handlers [7], and sewage workers [8,9].

HAV infection has long been discussed as an occupational health concern among municipal waste collection workers [2,10,11,12,13]. These workers operate primarily in urban streets, which are dynamic and often unpredictable environments, and where exposure to biological hazards is frequent. In Italy, their tasks include door-to-door waste collection, street cleaning, and the management of recycling centers. These activities may involve manual or mechanized handling of waste and contact with decomposing organic waste, bioaerosols, and contaminated materials. Municipal waste collection workers are potentially exposed to different infectious agents through mucocutaneous contact, inhalation, animal bites, insect stings, accidental ingestion, or injury from sharp, contaminated objects.

Early evidence from Italy [10] and Greece [11,12] reported elevated HAV seroprevalence rates in this population. Studies conducted in Thailand [14] also supported the link between occupational exposure and HAV risk. However, a cross-sectional study by Rachiotis et al. [13], based on a sample of 50 participants, did not confirm a significantly higher HAV seroprevalence among waste collectors compared to non-exposed workers. This discrepancy highlights the complexity of assessing occupational HAV risk and suggests that contextual factors may influence outcomes. The persistence of this issue across different countries and time periods highlights the need for updated epidemiological evidence to support occupational health surveillance and better inform public health interventions, including vaccination initiatives, in potentially exposed workers.

To our knowledge, the most recent Italian study addressing this topic was conducted in 1985 by Corrao et al. [10] and was not focused on HAV and waste collection workers. Therefore, the present study aims to contribute to the international debate on the occupational risks associated with HAV biological hazards in this specific workforce, providing updated epidemiological evidence from Italy. Using occupational health surveillance data, we investigated HAV seroprevalence and vaccination history among municipal waste collection workers across Sardinia and explored associated demographic and occupational risk factors. This assessment aims not only to offer insights into the extent to which waste collection activities may contribute to HAV transmission but also to provide epidemiological evidence to inform targeted public health interventions and preventive strategies, including measures to improve vaccination uptake, in this specific workforce and in similar occupational settings.

## 2. Materials and Methods

**Figure 1:**
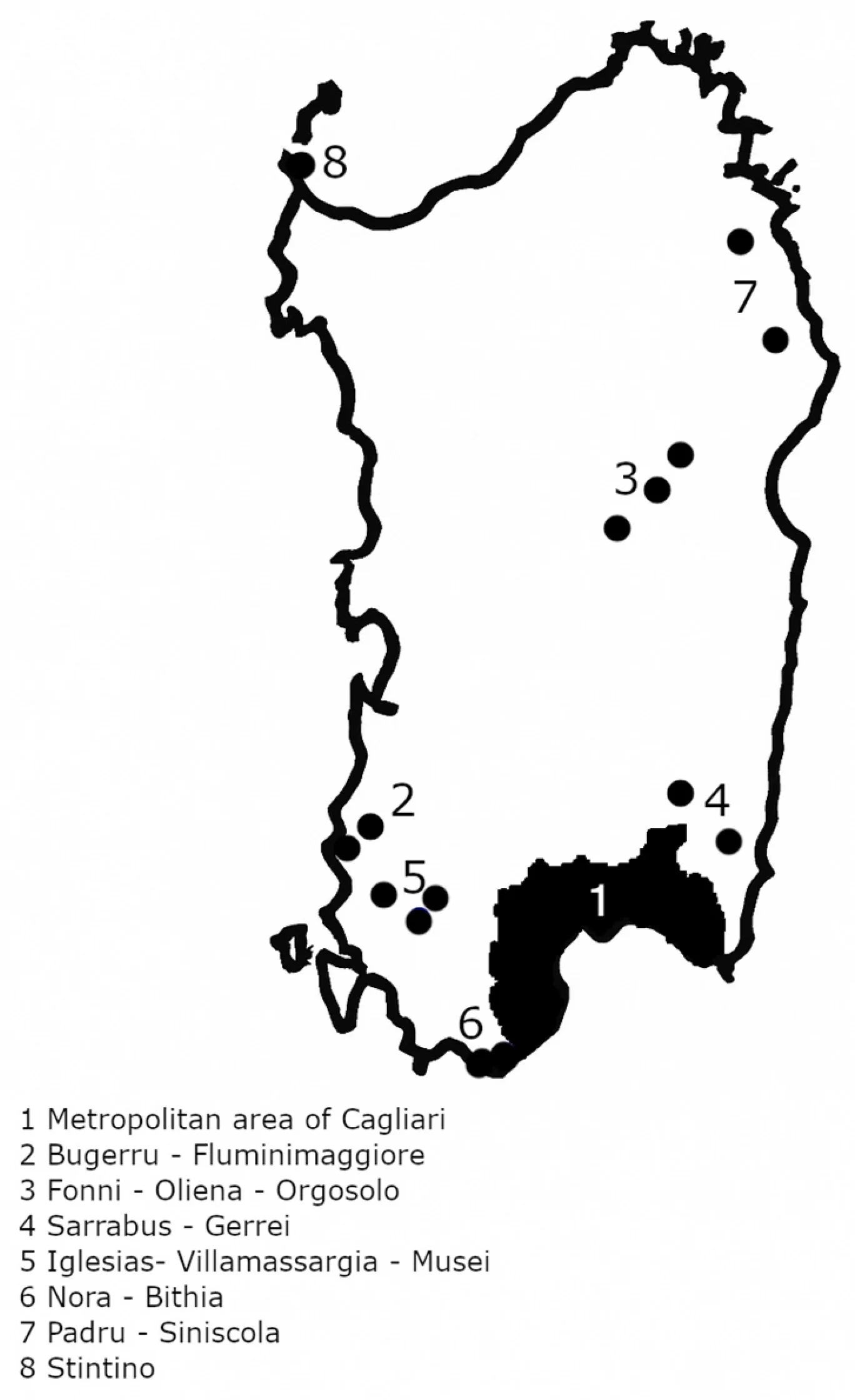
Worksites. Municipalities/areas where the waste collection workers included in the study population operated.

A retrospective cross-sectional study was conducted among municipal waste collection workers employed by three medium-to-large waste management companies operating in Sardinia (Italy), operating in the following municipalities/areas: Metropolitan city of Cagliari, Buggerru and Fluminimaggiore, Fonni–Oliena–Orgosolo, Gerrei, Iglesias–Villamassargia–Musei, Nora–Bithia, Padru–Siniscola, and Stintino (Figure 1).

These companies were selected through a convenience sampling strategy based on their established health surveillance agreements with the occupational health physician, allowing for a representative coverage of diverse operational environments across Sardinia, including metropolitan areas, small towns, and rural settings (categorized across 8 operational locations) [15]. The total eligible workforce across the three companies during the study period comprised all employees undergoing mandatory routine occupational health surveillance under Article 41 of Legislative Decree 81/2008 [16]. Data were collected between January 2017 and July 2024. Inclusion criteria were: (i) male and female workers aged ≥18 years employed as municipal waste collectors in one of the three participating companies during the study period; (ii) completion of routine occupational health assessments; (iii) provision of signed written informed consent authorizing the use of their personal and clinical data in an anonymized and aggregated form for scientific research purposes, in accordance with European and Italian data protection regulations (GDPR). Exclusion criteria were: (i) workers with incomplete medical health records lacking core demographic or serological data; (ii) administrative or office personnel without active occupational exposure to waste or sewage; (iii) workers who withheld consent for the scientific use of their anonymized data. Under Italian occupational health law (D.Lgs. 81/2008), undergoing medical examination and health surveillance is a statutory requirement for workers exposed to biological risks. However, consenting to the use of health records for scientific research was strictly voluntary. During the routine occupational health visit, workers were informed about the scientific scope of the data collection and signed a written privacy and data processing consent form. Declining consent for research purposes carried no impact on the worker’s occupational fitness-for-work evaluation, routine health surveillance, or employment status. Out of 338 eligible waste collection workers invited for health surveillance during the study timeframe, 326 provided informed consent and had complete clinical and serological records, resulting in a participation rate of 96.4% (326/338). Data from 12 workers (3.6%) were excluded prior to analysis: 9 due to incomplete medical histories or missing serological testing, and 3 due to withheld consent for the research use of their data. No worksite-based sampling or selection procedure was applied at any stage. Because missing data accounted for less than 5% of the total eligible cohort, a complete-case analysis was performed without applying imputation methods; subjects with a missing value on a given variable were excluded only from analyses involving that variable (e.g., years of service data were unavailable for three workers; see Table 1), while being retained in all other analyses. As the study sample coincided with the full occupational cohort under mandatory surveillance, no a priori sample size calculation was used to guide recruitment; for reference, applying Cochran’s formula for a finite population, assuming an expected HAV seroprevalence of 30% (based on data in the literature for high-risk occupational groups), a 95% confidence level (Z = 1.96), and a 5% margin of error, the minimum sample required for prevalence estimation would be 323 subjects, closely matched by the final sample (N = 326). A post hoc power analysis was additionally performed on the main between-group comparisons (Cohen’s w for chi-square tests; rank-biserial correlation converted to Cohen’s d for Mann–Whitney U tests), using the same Bonferroni-corrected alpha thresholds; results are reported in Section 3 and discussed as a limitation where power was insufficient.

During the initial health assessment visit, the occupational health physician compiled a standardized medical record documenting personal demographic data, smoking habits, occupational history, clinical background, and self-reported vaccination status. Blood samples were collected once per worker to assess HAV seroprevalence via serum anti-HAV antibody testing (total IgG). The calculated seroprevalence reflects the overall 8-year study period and is not intended to analyze temporal trends. The main study variables recorded included age at sampling (analyzed as a continuous variable and dichotomized into ≤48 and >48 years based on the sample median cut-off), sex, smoking habits (smoker, non-smoker, or former smoker), years of service (analyzed as a continuous variable and dichotomized into <9 and ≥9 years based on the sample median cut-off), work setting (categorized across 8 locations: metropolitan, rural, or small town), self-reported vaccination status, and anti-HAV serological result. These cut-offs were applied solely to categorize continuous data for subgroup comparisons and did not represent inclusion limits or restrict the cohort analysis.

Participant characteristics were summarized using absolute counts (*n*) and relative frequencies (%) for categorical variables, and mean with standard deviation (SD) for continuous variables. The normality of continuous variables was assessed using the Shapiro–Wilk test; as normality was violated (see Supplementary Materials, Table S1), non-parametric Mann–Whitney U tests were applied for continuous group comparisons. Chi-square (X^2^) tests were used for categorical variables. To adjust for multiple comparisons, a Bonferroni correction was applied, setting statistical significance at *p* < 0.01 for X^2^ tests and *p* < 0.025 for Mann–Whitney U tests. Multivariable logistic regression was used to identify independent predictors of HAV seropositivity; no adjustment to alpha was made for the multivariable model, as all variables were analyzed simultaneously. Given that HAV seropositivity was not a rare outcome in this sample (44.2%), a modified Poisson regression model with robust (sandwich) variance estimation was additionally fitted, using the same predictors as the logistic regression model, to estimate prevalence ratios (PRs) as a complementary measure of association less prone to overestimation than the odds ratio. To address potential confounding between age and years of service (Pearson’s r = 0.449) and to avoid the loss of information associated with arbitrary categorical cut-offs, a sensitivity analysis was conducted in which age and years of service were entered into the logistic regression model as continuous variables (per 10-unit increase), both separately and jointly. The Generalized Variance Inflation Factor (GVIF) was computed to assess collinearity among predictors, as this measure appropriately accounts for multi-degree-of-freedom categorical terms (smoking habits and work setting) unlike the conventional VIF. An age × years-of-service interaction term was tested via a likelihood ratio test to assess potential effect modification. Non-linearity in the log-odds of the continuous age and years-of-service effects was assessed by adding quadratic terms to the model and comparing model fit via a likelihood ratio test.

All statistical analyses were conducted using JASP (Version 0.19.2, JASP Team, 2023, Amsterdam, The Netherlands).

## 3. Results

### 3.1. Descriptive Analysis

Male workers accounted for 99% of the sample (*n* = 323), while only 0.9% (*n* = 3) were female. The mean age at the time of blood sampling was 46.44 years (SD = 9.2), with a median age of 48 years and an age range between 20 and 65 years. Regarding lifestyle habits, 59.2% of participants reported being current or former smokers during the initial health assessment. The mean of years of service was 10.28 years (SD = 10.3), with a median of 9 years and a range from 0 to 42 years.

Serological testing revealed that 144 workers (44.2%) were positive for anti-HAV antibodies, indicating either past infection or vaccination, while 182 workers (55.8%) were seronegative, suggesting no prior exposure or immunization. Only three workers (0.9%) reported having received the HAV vaccine, highlighting a very low self-reported vaccination coverage within the cohort.

### 3.2. Univariate Analysis

The X^2^ test was used to evaluate whether there were statistically significant differences in the distribution of HAV seropositivity across categorical variables. Given the limited representation of female subjects in the sample (*n* = 3), we restricted the analysis of sex to descriptive statistics.

**Figure 2:**
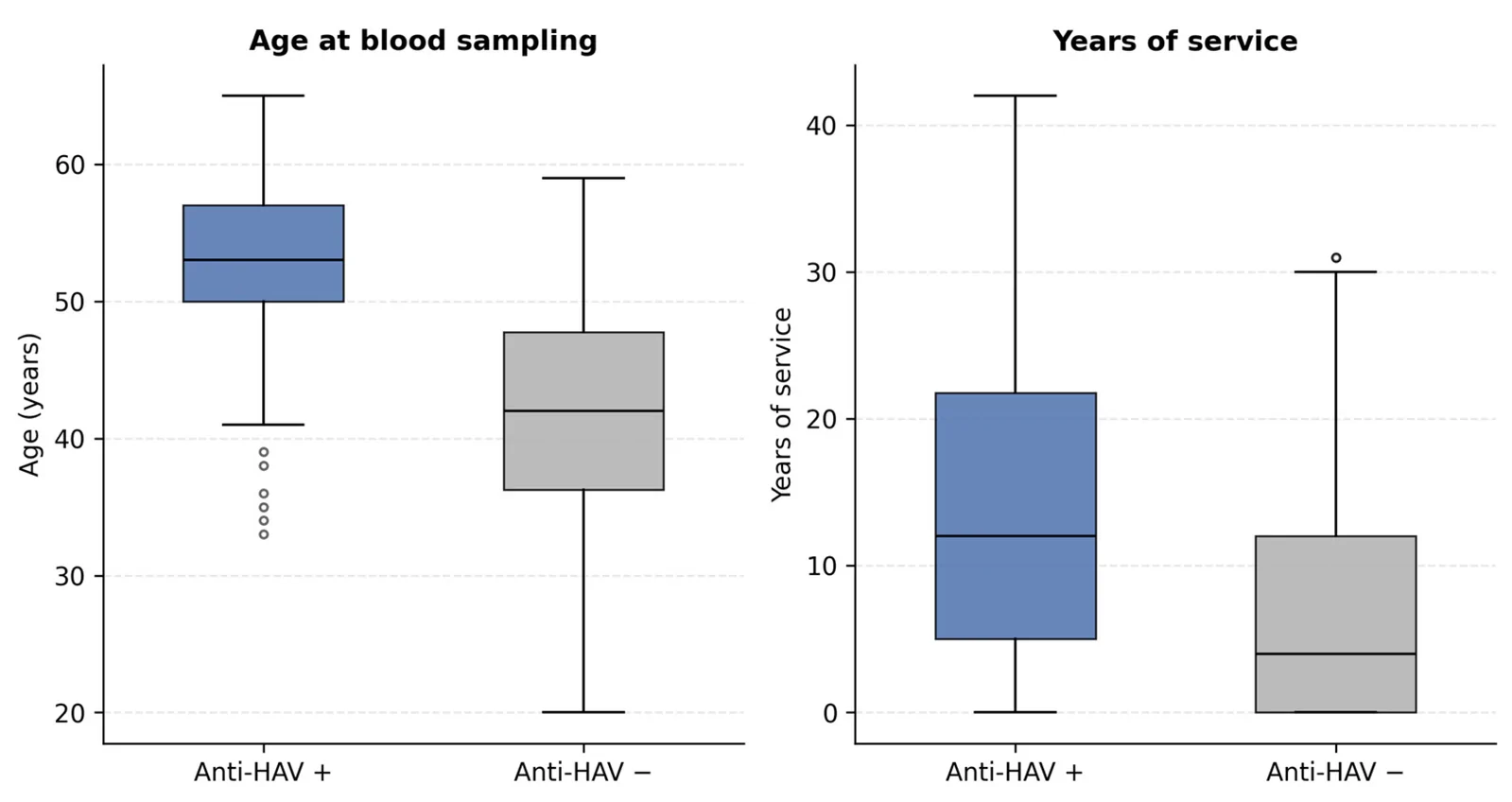
Distribution of age at blood sampling and years of service by HAV serological status. Boxes represent the median and interquartile range (IQR, Q1–Q3) as reported in Table 1.

A statistically significant difference was observed in the distribution of HAV seropositivity (Figure 2) between workers aged ≥ 48 years and those younger (X^2^ = 105.8; df = 1; *p* < 0.001). The median age of HAV-positive individuals was 53 years (IQR = 50–57), while the median age of HAV-negative individuals was 42 (IQR = 36.3–47.8). A Mann–Whitney U test was conducted to compare age at the time of sampling between the two independent groups. The results revealed a statistically significant difference (U = 3063.500; *p* < 0.001). The effect size, calculated using the rank-biserial correlation, was substantial (r(rb) = −0.766; SE = 0.064), suggesting a strong negative association between group and age. This implies that the HAV-positive group was consistently older than the other, with minimal overlap in age distributions. The distribution of HAV seropositivity differed significantly between workers with ≥9 years of service and those with less experience (X^2^ = 23.16; df = 1; *p* < 0.001). The median of the years of service among HAV-positive workers was 12 (IQR = 5–21.8), compared to 4 years (IQR = 0–12) among HAV-negative workers. The Mann–Whitney U test revealed a statistically significant difference (*U* = 8459.000; *p* < 0.001), indicating that HAV-positive individuals had a different distribution of service duration compared to HAV-negative individuals. The effect size, expressed as rank-biserial correlation, was moderate (r(rb) = −0.342; SE = 0.065), with a 95% confidence interval ranging from −0.449 to −0.225. The negative direction of the correlation indicates that HAV-positive individuals tended to have more years of service than HAV-negative individuals, suggesting a possible association between increased years of service and HAV seropositivity.

No statistically significant difference was found in HAV seropositivity between smokers, former smokers, and non-smokers (X^2^ = 4.07; df = 2; *p* = 0.131), nor between professionals from different work settings (X^2^ = 8.60; df = 7; *p* = 0.283). See Table 1 for details.

### 3.3. Multivariate Analysis

To further explore the potential associations suggested by X^2^ and Mann–Whitney U test results, and refine our analysis, a binary logistic regression model was constructed. The full model (M_1_), which included age group, years of service, smoking habits, and worksite, significantly improved model fit compared to the null model (M_0_) (ΔΧ^2^ = 130.642; df = 11; *p* < 0.001). Model performance indicators were acceptable, with McFadden R^2^ = 0.295 and Nagelkerke R^2^ = 0.446 (Table 2).

Age < 48 years was strongly associated with reduced odds of HAV seropositivity (OR = 0.068; 95% CI: 0.037–0.124; *p* < 0.001). Conversely, workers aged 48 years and older had approximately 14.7 times higher odds of HAV seropositivity compared to younger workers. This highlights a strong association between older age and increased likelihood of past HAV exposure or infection. Years of service ≥ 9 were significantly associated with increased risk (OR = 2.773; 95% CI: 1.507–5.104; *p* = 0.001), suggesting that workers with less than 9 years of service had 64% lower odds of HAV seropositivity (inverse OR = 0.36). Worksite 7 showed statistically significant increased odds (OR = 3.463; 95% CI: 1.007–11.912; *p* = 0.049), while worksite 6 had an increased odds ratio (OR = 1.887) that did not reach statistical significance (*p* = 0.134). Smoking habits and other worksites did not show significant associations with HAV seropositivity. See Table 3 for details.

No evidence of multicollinearity was found among the independent variables included in the logistic regression model (see Table 4). The ROC curve (Supplementary Materials, Figure S1) demonstrated good discriminative ability of the model, with an area under the curve AUC = 0.843. Residual plots (Supplementary Materials, Figure S2) showed a symmetric distribution across predicted probabilities, suggesting adequate model calibration and no major specification errors. The binary categorization of age (<48 vs. ≥48 years) and years of service (<9 vs. ≥9 years) was retained in the final model. Residual diagnostics did not suggest the need for more granular stratification, supporting the adequacy of these simplified groupings within the model.

#### 3.3.1. Modified Poisson Regression with Robust Variance

To complement the logistic regression findings, given the non-rare nature of HAV seropositivity in this sample, a modified Poisson regression model with robust variance was fitted to estimate prevalence ratios (PRs). Findings were directionally consistent with the logistic regression model: age < 48 years was associated with a markedly lower prevalence of seropositivity (PR = 0.230; 95% CI: 0.158–0.334; *p* < 0.001), while years of service ≥ 9 years (PR = 1.538; 95% CI: 1.177–2.008; *p* = 0.002) and worksite 7 (PR = 1.613; 95% CI: 1.183–2.199; *p* = 0.003) were associated with higher prevalence. Smoking habits and the remaining worksites were not significantly associated with HAV seropositivity, consistent with the logistic regression results (Table 5). As expected, PR estimates were attenuated relative to the corresponding OR, reflecting the well-documented tendency of the odds ratio to overstate the relative risk when the outcome is common.

#### 3.3.2. Sensitivity Analysis: Age and Years of Service as Continuous Variables

Age and years of service were moderately correlated (Pearson’s r = 0.449). When entered separately into the multivariable model (continuous, per 10-unit increase, adjusted for smoking and work setting), both age (OR = 13.10; 95% CI: 7.42–23.14; *p* < 0.001) and years of service (OR = 1.99; 95% CI: 1.52–2.59; *p* < 0.001) were strongly associated with HAV seropositivity. However, when entered jointly, the association with years of service was substantially attenuated and no longer statistically significant (OR = 1.14; 95% CI: 0.81–1.59; *p* = 0.452), while the association with age remained essentially unchanged (OR = 12.30; 95% CI: 6.85–22.11; *p* < 0.001) (Table 6). GVIF values for all model terms were low (age: 1.14; years of service: 1.19; smoking: 1.02; work setting: 1.02, on the adjusted GVIF^1/2df scale), indicating that this attenuation reflects confounding by age rather than problematic collinearity. No significant age × years-of-service interaction was detected (likelihood ratio test, *p* = 0.42). Quadratic terms for both age and years of service were borderline non-significant (*p* = 0.06 and *p* = 0.07, respectively), and visual inspection of binned prevalence by age and years of service (Supplementary Materials Figure S3) suggested a pattern broadly consistent with linearity on the logit scale.

### 3.4. Post Hoc Power Analysis

Post hoc power analysis showed that the study sample provided adequate power (>0.98) to detect the observed associations between HAV seropositivity and age (Cohen’s w = 0.570 and power > 0.999 for the chi-square test; d ≈ 2.38 and power > 0.999 for the Mann–Whitney U test) and years of service (Cohen’s w = 0.268 and power = 0.987 for the chi-square test; d ≈ 0.73 and power > 0.999 for the Mann–Whitney U test). Conversely, power was limited for smoking habits (w = 0.112; power = 0.209) and work setting (w = 0.162; power = 0.302), indicating that the sample was underpowered to detect small effect sizes for these variables at the corrected significance threshold.

## 4. Discussion

The main objective of this study was to evaluate hepatitis A virus (HAV) seroprevalence, self-reported vaccination history, and associated occupational and sociodemographic risk factors among municipal waste collection workers in Sardinia, Italy. The study revealed a notably high seven-year (2017–2024) cumulative seroprevalence of HAV among waste collection workers in Sardinia, with 44.2% testing positive for anti-HAV antibodies. Statistical analysis indicated that older age and longer occupational exposure were significantly associated with HAV seropositivity. Specifically, workers aged 48 and above, as well as those with at least nine years of service, had a higher likelihood of testing positive. These associations, initially suggested by univariate analysis, were confirmed in the multivariate logistic regression model. In contrast, younger workers and those with fewer years of service were significantly less likely to show evidence of past HAV infection, reinforcing the importance of early preventive measures in this occupational group. It should be noted that, because HAV seropositivity was a relatively common outcome in this cohort (44.2%), the odds ratios derived from the logistic regression model likely overestimate the true strength of association compared to the prevalence ratio. The complementary Poisson regression analysis with robust variance confirmed the direction and statistical significance of the main associations observed in the categorical model, while providing more directly interpretable effect size estimates; as detailed below; however, the independent contribution of years of service was substantially attenuated once age was modeled as a continuous variable. Furthermore, a sensitivity analysis using age and years of service as continuous variables (Section 3.3.2) showed that the association with years of service was substantially attenuated and lost statistical significance once age was properly accounted for, while age remained strongly and independently associated with seropositivity; given the low GVIF values observed, this pattern reflects confounding by age rather than collinearity. This finding moderates our interpretation: rather than reflecting a dose-dependent effect of cumulative occupational exposure, the observed pattern more plausibly reflects birth-cohort differences in lifetime HAV exposure risk, consistent with the generally higher HAV exposure among older birth cohorts in Italy prior to the widespread improvement in sanitation and hygiene standards. This pattern aligns with trends reported in general population studies, where HAV prevalence increases with age [17], likely reflecting both environmental and behavioral factors that accumulate over time. To further contextualize this age-related pattern, we compared our findings with published seroprevalence data from the general population in Sardinia and Italy. In a hospital-based sample from Cagliari (Sardinia), Campagna et al. [17] reported an overall anti-HAV seroprevalence of 74.6%, with markedly lower values in subjects younger than 40 years (17.5%) and under 30 years (8.9%), reflecting a substantial decline in HAV circulation over recent decades. At the national level, a hospital-based serosurvey conducted across 18 Italian regions reported a crude anti-HAV seroprevalence of 32% (age-adjusted 60.1%), with a marked birth-cohort effect and a north–south gradient (55% in northern Italy vs. 68% in southern/insular regions, including Sardinia) [18]. The age distribution observed in our cohort (15.5% seropositivity among workers < 48 years vs. 72.1% among those ≥48 years) is broadly consistent with these regional and national patterns, supporting the interpretation that historical, non-occupational background exposure—rather than a cumulative occupational dose–response effect—accounts for a substantial share of the anti-HAV positivity observed among older workers. Overall, the results align with previous international studies on waste workers, which also reported cumulative seroprevalence of anti-HAV antibodies. For instance, Rachiotis et al. [2] reported a 40% prevalence among Greek municipal waste collectors, while earlier studies documented even higher rates—up to 62.5% [12].

Workers at worksite 7 (Padru–Siniscola) showed a higher prevalence ratio of HAV seropositivity relative to the reference site (PR = 1.613; 95% CI: 1.183–2.199; *p* = 0.003), while the corresponding odds ratio from the logistic regression model was less precisely estimated (OR = 3.463; 95% CI: 1.007–11.912; *p* = 0.049), with a lower confidence bound close to the null. This difference in precision between the two models reflects the small sample size at this site (*n* = 18), and the effect size for worksite 7 should accordingly be regarded as more robust in the Poisson model than in the logistic model, where the wide confidence interval indicates limited reliability of the point estimate. Moreover, the omnibus test for work setting was not statistically significant (χ^2^ = 8.60; df = 7; *p* = 0.283), indicating limited overall evidence of a worksite effect across the cohort. We therefore consider the worksite 7 finding exploratory and hypothesis-generating rather than confirmatory, and it should not be interpreted as established evidence of geographic variability in HAV risk. Furthermore, any differences observed between worksites may reflect organizational or occupational characteristics—such as local operational procedures, team composition, or waste handling practices—rather than geography itself, and our data cannot distinguish between these explanations. We were unable to identify publicly available, municipality-level HAV case surveillance data to determine whether the general population in the Padru–Siniscola area experienced a correspondingly elevated HAV incidence during the study period, as Italian national surveillance data (SEIEVA) are reported at the regional rather than municipal level and no documented outbreak in this specific area was identified in the sources consulted. This limitation is compounded by the fact that individual-level residence data were not available in this study, preventing any individual-level linkage between workers’ place of residence and local community HAV notifications. Access to local health authority records on general-population HAV notifications in this area would help clarify whether this pattern reflects a broader community-level epidemiological signal or is specific to occupational exposure. Further studies with larger samples and more detailed environmental assessments are needed to clarify the role of geographic and procedural determinants in HAV exposure.

Smoking during work may theoretically increase the risk of oro-fecal transmission if performed with contaminated hands, especially in environments with poor hygiene conditions, a behavioral pathway previously suggested as a potential risk factor for HAV exposure among waste handlers [2]. From a safety perspective, this hand-to-mouth mechanism underlines the value of targeted preventive interventions, where occupational health physicians inform workers about these risks during health surveillance and safety training, reinforcing proper hand hygiene and glove removal prior to smoking breaks. However, in our study, smoking habits were not significantly associated with HAV seropositivity, neither in univariate nor multivariate analysis. This suggests that tobacco use, as an isolated lifestyle factor, may not play a major role in HAV transmission within this occupational group. It must be noted that behavioral factors such as hand hygiene and food consumption practices during work shifts were not assessed, limiting the ability to evaluate their potential contribution to HAV exposure.

Interestingly, only 0.9% of the workers reported having received HAV vaccination, despite national recommendations targeting high-risk groups, such as waste handlers. The extremely low self-reported vaccination coverage highlights a critical gap in the implementation of preventive strategies, including vaccination, for occupationally exposed workers. This gap between recommendations and actual coverage reflects crucial challenges in adult vaccination programs, which may include difficulties in access, lack of awareness, and insufficient integration of occupational health and public health systems. Exposure and lack of vaccination, combined with limited risk awareness and training, may contribute to the high infection rates observed. Consistent with this, Mol et al. [19] emphasized the importance of incorporating workers’ perceptions into the design of preventive measures, showing that awareness and engagement are key to improving safety outcomes in waste collection settings. Future research should explore the lived experiences and perceptions of risk among Sardinian waste collection workers to better understand vulnerabilities and guide more effective and context-sensitive public health interventions.

All findings should be interpreted within the specific occupational and geographic context and in light of the methodological limitations: (i) cross-sectional design and single-time testing per participant, limiting the ability to assess changes over time and preventing determination of when HAV infection occurred relative to occupational entry; consequently, associations with years of service cannot be interpreted as evidence of a cumulative occupational dose–response effect, since workers with longer service histories may have acquired infection before starting this occupation; (ii) absence of a control group: HAV seropositivity may also reflect non-occupational exposures and individual or contextual characteristics (e.g., residence, hygiene habits, and birth cohort), and its attribution to occupational exposure to waste cannot be confirmed in the absence of a sociodemographically comparable, non-exposed comparator group; (iii) self-reported vaccination status: subject to recall and under-reporting bias, meaning that undetected vaccination cannot be fully excluded and may partly account for observed seropositivity; however, given the extremely low overall number of reported vaccinated individuals, potential reporting inaccuracies are unlikely to affect the main findings substantially; moreover, adult HAV vaccination data in Italy are fragmented and lack a centralized national registry; (iv) low representation of female workers, which prevented sex-based sub-analyses; notably, this reflects the structural workforce composition in the regional waste collection sector rather than a sampling bias; (v) unmeasured variables: several potentially relevant factors (e.g., exact residence, education level, health literacy, personal hygiene habits, nationality, use of personal protective equipment, and pre-employment health status) were not systematically collected during occupational health assessments; standardizing these variables in future surveillance will enable a fuller characterization of HAV risk determinants; (vi) lack of local surveillance granularity: publicly available, municipality-level HAV data were inaccessible to determine whether the general population in areas with elevated worker seropositivity (e.g., Padru–Siniscola) experienced a higher HAV incidence, as Italian national surveillance data (SEIEVA) are reported only at the regional level; (vii) worksite clustering considerations: although worksite was included as a fixed effect to account for between-site differences, residual within-site clustering of unmeasured factors cannot be fully excluded; a random-effects model was not applied due to the limited number of worksites (*n* = 8), which precludes reliable estimation of between-cluster variance. Future research should prioritize longitudinal designs with repeated, dated serological testing and the recruitment of a matched, non-exposed general population comparison group, especially given the lack of national age-stratified HAV seroprevalence data. A post hoc power analysis confirmed that the sample size was fully adequate to detect associations between HAV seropositivity, age, and years of service. However, statistical power was limited for comparisons involving smoking habits and work setting; thus, non-significant findings for these variables should be interpreted as inconclusive rather than definitive evidence of no effect. Overall, the results highlight the need for targeted preventive measures for municipal waste collection workers in Sardinia, including improved access to HAV vaccination, hygiene education, and risk-awareness initiatives. Integrating occupational health surveillance with broader public health strategies remains essential to mitigate biological risks in vulnerable worker populations.

## 5. Conclusions

In conclusion, this study provides valuable epidemiological insights suggesting that older age, longer years of service, and certain worksite conditions are associated with increased HAV seropositivity among municipal waste collection workers in Sardinia, Italy. However, the association between age and seropositivity must be interpreted considering historical epidemiological trends, as older workers likely experienced higher non-occupational exposure in past decades when community HAV circulation was substantially higher. These findings suggest that municipal waste collection workers may represent an occupational group characterized by substantial HAV exposure and infection burden and highlight the importance of integrating occupational health surveillance with public health strategies to mitigate biological risks in vulnerable worker populations. Furthermore, the extremely low self-reported vaccination history highlights both the need and the opportunity for targeted public health interventions aimed at improving vaccination uptake, awareness, and engagement among waste collection workers. Occupational health surveillance should be strengthened to include routine HAV screening and vaccination programs for high-risk groups, while periodic population-level seroprevalence assessment remains essential to support regular epidemiological monitoring.

## Figures and Tables

**Table 1:** Univariate analysis of anti-HAV seropositivity by sex, age, years of service, and smoking habits.

Variables	Anti-HAV+	Anti-HAV−	*p*	Test
**Sex**			N/A	--
Male	143 (43.9%)	180 (55.2%)		
Female	1 (0.3%)	2 (0.6%)		
**Age at the time of blood sampling**			<0.001	Chi-square test
<48 years	25 (7.7%)	136 (41.7%)		
≥48 years	119 (36.5%)	46 (14.1%)		
Median age (IQR)	53 (50–57)	42 (36.3–47.8)	<0.001	Mann–Whitney U test
**Years of service**			<0.001	Chi-square test
<9 years	48 (14.7%)	110 (33.7%)		
≥9 years	94 (28.8%)	71 (21.8%)		
Median years of service (IQR)	12 (5–21.8)	4 (0–12)	<0.001	Mann–Whitney U test
**Smoking habits**			0.131	Chi-square test
Non-smoker	63 (19.3%)	70 (21.5%)		
Former smoker	37 (11.3%)	37 (11.3%)		
Smoker	44 (13.5%)	75 (23%)		
**Work setting**			0.283	Chi-square test
1 (n. 130)	63 (19.4%)	67 (20.7%)		
2 (n. 12)	3 (0.9%)	9 (2.8%)		
3 (n. 23)	9 (2.8%)	14 (4.3%)		
4 (n. 11)	6 (1.8%)	5 (1.5%)		
5 (n. 58)	23 (7.1%)	35 (10.8%)		
6 (n. 52)	23 (7.1%)	29 (8.9%)		
7 (n. 18)	11 (3.4%)	7 (2.2%)		
8 (n. 22)	6 (1.8%)	16 (5%)		

Note: N/A (Not applicable); Work settings: 1—Metropolitan city of Cagliari; 2—Buggerru and Fluminimaggiore; 3—Fonni–Oliena–Orgosolo; 4—Gerrei; 5—Iglesias–Villamassargia–Musei; 6—Nora–Bithia; 7—Padru–Siniscola; 8—Stintino. Years of service data were unavailable for three workers. IQR = interquartile range.

**Table 2:** Model summary—anti-HAV.

Model	Deviance	AIC	BIC	df	ΔΧ^2^	*p*	McFadden R^2^	Nagelkerke R^2^	Tjur R^2^	Cox & Snell R^2^
M_0_	443.1	445.053	448.830	322			0.000		0.000	
M_1_	312.4	336.410	381.742	311	130.642	<0.001	0.295	0.446	0.365	0.333

Note. M_1_ includes age at test, years of service, smoking habits, and work setting.

**Table 3:** Logistic regression coefficients for HAV seropositivity.

Model	Variable	Estimate	Standard Error	Odds Ratio	95% CI Lower	95% CI Upper	*p*
M_0_	(Intercept)	−0.243	0.112	0.785	0.630	0.977	0.030
M_1_	(Intercept)	0.399	0.377	1.490	0.712	3.120	0.290
	Age at test (<48)	−2.690	0.305	0.068	0.037	0.124	**<0.001**
	Years of service (≥9)	1.020	0.311	2.773	1.507	5.104	**0.001**
	Smoking habits (smoker)	−0.214	0.331	0.807	0.422	1.545	0.518
	Smoking habits (former smoker)	−0.369	0.364	0.692	0.339	1.411	0.311
	Work setting (2)	−0.198	0.900	0.821	0.141	4.791	0.826
	Work setting (3)	−0.598	0.584	0.550	0.175	1.728	0.306
	Work setting (4)	−0.008	0.754	0.992	0.226	4.347	0.991
	Work setting (5)	0.348	0.426	1.417	0.614	3.268	0.414
	Work setting (6)	0.635	0.424	1.887	0.822	4.335	0.134
	Work setting (7)	1.242	0.630	3.463	1.007	11.912	**0.049**
	Work setting (8)	−0.269	0.632	0.764	0.221	2.638	0.671

Note: M_0_ = null model; M_1_ = full model. Significant *p*-values are reported in bold. Reference categories are: age ≥ 48 years, years of service < 9 years, non-smoker for smoking habits, and work setting (1) for work settings.

**Table 4:** Multicollinearity diagnostics.

	Tolerance	VIF
Age at test	0.893	1.119
Years of service	0.826	1.210
Smoking habits	0.929	1.077
Work setting	0.744	1.344

Note: VIF = variance inflation factor.

**Table 5:** Modified Poisson regression with robust variance: prevalence ratios (PRs) for HAV seropositivity. Ref. = reference.

Variable	PR	95% CI	*p*
Age < 48 years (ref. ≥ 48)	0.230	0.158–0.334	<0.001
Years of service ≥ 9 years (ref. < 9)	1.538	1.177–2.008	0.002
Smoker (ref. non-smoker)	0.922	0.727–1.169	0.503
Former smoker (ref. non-smoker)	0.883	0.692–1.127	0.318
Worksite 2 (ref. worksite 1)	0.863	0.529–1.409	0.556
Worksite 3 (ref. worksite 1)	0.797	0.526–1.208	0.285
Worksite 4 (ref. worksite 1)	1.014	0.563–1.828	0.962
Worksite 5 (ref. worksite 1)	1.138	0.824–1.572	0.432
Worksite 6 (ref. worksite 1)	1.327	0.957–1.839	0.090
Worksite 7 (ref. worksite 1)	1.613	1.183–2.199	0.003
Worksite 8 (ref. worksite 1)	0.882	0.463–1.683	0.704

**Table 6:** Sensitivity analysis: age and years of service (continuous, per 10-unit increase) entered separately vs. jointly.

Variable	Model	OR	95% CI	*p*
Age (per 10 years)	Alone	13.10	7.42–23.14	<0.001
Age (per 10 years)	Adjusted for years of service	12.30	6.85–22.11	<0.001
Years of service (per 10 years)	Alone	1.99	1.52–2.59	<0.001
Years of service (per 10 years)	Adjusted for age	1.14	0.81–1.59	0.452

## Data Availability

All data generated or analyzed during this study are available from the corresponding author upon request.

## Supplementary Materials

The following supporting information can be downloaded at https://www.mdpi.com/article/10.3390/vaccines14090767/s1, Table S1: Assumption checks, Figure S1: ROC curve, Figure S2: Residual plots. Figure S3: Observed HAV seroprevalence by age and years of service.

## Abbreviations

The following abbreviations are used in this manuscript:

AUC	Area Under the Curve
CI	Confidence Interval
HAV	Hepatitis A Virus
IQR	Interquartile Range
OR	Odds Ratio
ROC	Receiver Operating Characteristic
SD	Standard Deviation
SEIEVA	Integrated Epidemiological Surveillance System for Acute Viral Hepatitis
VIF	Variance Inflation Factor
X^2^	Chi-square Test
