# Supplementary material for: High Hepatitis A Virus Seroprevalence and Low Self-Reported Vaccination Coverage Among Waste Collection Workers in Sardinia, Italy: A Retrospective Cross-Sectional Study
Source: Vaccines (Basel). 2026 Sep 2;14(9):767. doi: 10.3390/vaccines14090767 (PMC13611448; doi:10.3390/vaccines14090767)
Supplement: Supplementary file 1 [file vaccines-14-00767-s001.zip › vaccines-4494939-supplementary.pdf]

Supplemental Materials

Table S1. Assumption Checks.

Test of Normality (Shapiro-Wilk)

Residuals	W	p
Age at time of test	0.979	< .001

Note. Significant results suggest a deviation from normality.

Test of Normality (Shapiro-Wilk)

Residuals	W	p
Years of service	0.928	< .001

Note. Significant results suggest a deviation from normality.

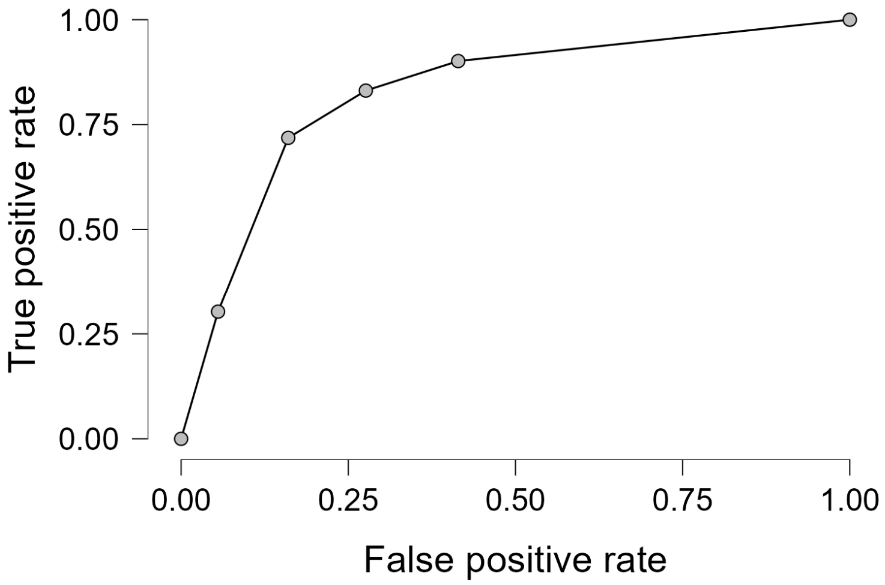

Figure S1. ROC curve.

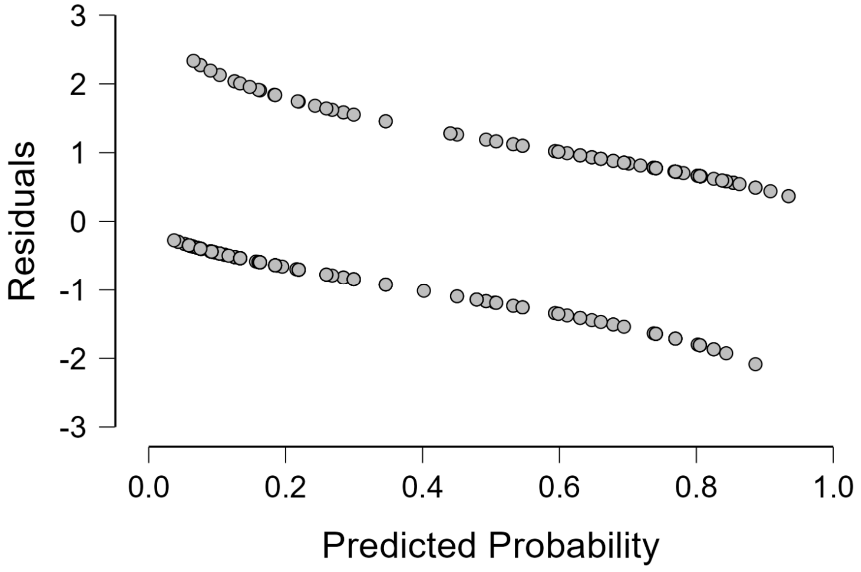

Figure S2. Residual plots.

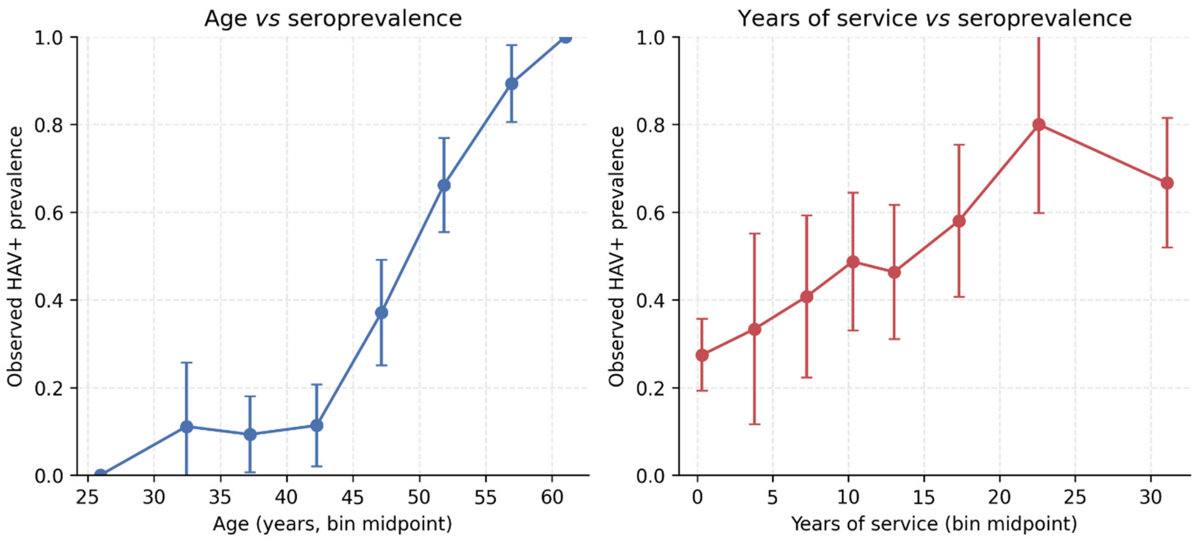

Figure S3. Observed HAV seroprevalence by age and years of service.
